# Chicken-Specific Kinome Array Reveals that *Salmonella enterica* Serovar Enteritidis Modulates Host Immune Signaling Pathways in the Cecum to Establish a Persistence Infection

**DOI:** 10.3390/ijms17081207

**Published:** 2016-07-27

**Authors:** Michael H. Kogut, Christina L. Swaggerty, James Allen Byrd, Ramesh Selvaraj, Ryan J. Arsenault

**Affiliations:** 1 Southern Plains Agricultural Resarch Center, United States Department of Agriculture, Agricultural Research Service, College Station, TX 77845, USA; mike.kogut@ars.usda.gov (M.H.K.); christi.swaggerty@ars.usda.gov (C.L.S.); allen.byrd@ars.usda.gov (J.A.B.); 2 Ohio Agricultural Research Center, The Ohio State University, Wooster, OH 44691, USA; selvaraj.7@osu.edu; 3 Department of Animal and Food Sciences, University of Delaware, Newark, DE 19716, USA

**Keywords:** *Salmonella*, kinome, interferon-γ, phospholipase c, JAK-STAT pathway

## Abstract

Non-typhoidal *Salmonella enterica* induces an early, short-lived pro-inflammatory response in chickens that is asymptomatic of clinical disease and results in a persistent colonization of the gastrointestinal (GI) tract that transmits infections to naïve hosts via fecal shedding of bacteria. The underlying mechanisms that control this persistent colonization of the ceca of chickens by *Salmonella* are only beginning to be elucidated. We hypothesize that alteration of host signaling pathways mediate the induction of a tolerance response. Using chicken-specific kinomic immune peptide arrays and quantitative RT-PCR of infected cecal tissue, we have previously evaluated the development of disease tolerance in chickens infected with *Salmonella enterica* serovar Enteritidis (*S*. Enteritidis) in a persistent infection model (4–14 days post infection). Here, we have further outlined the induction of an tolerance defense strategy in the cecum of chickens infected with *S*. Enteritidis beginning around four days post-primary infection. The response is characterized by alterations in the activation of T cell signaling mediated by the dephosphorylation of phospholipase c-γ1 (PLCG1) that inhibits NF-κB signaling and activates nuclear factor of activated T-cells (NFAT) signaling and blockage of interferon-γ (IFN-γ) production through the disruption of the JAK-STAT signaling pathway (dephosphorylation of JAK2, JAK3, and STAT4). Further, we measured a significant down-regulation reduction in IFN-γ mRNA expression. These studies, combined with our previous findings, describe global phenotypic changes in the avian cecum of *Salmonella* Enteritidis-infected chickens that decreases the host responsiveness resulting in the establishment of persistent colonization. The identified tissue protein kinases also represent potential targets for future antimicrobial compounds for decreasing *Salmonella* loads in the intestines of food animals before going to market.

## 1. Introduction

The Centers for Disease Control and Prevention continues to address multistate foodborne outbreaks that have impacted the health of the nation over the last 10 years [1]. One area of concern is the need for reduction of *Salmonella* as a foodborne pathogen. Despite control efforts that cost over a half a billion dollars annually, foodborne illnesses due to *Salmonella* continue to impact the consumer. Poultry are commonly identified as a major source of *Salmonella*. Asymptomatic carrier states are poorly understood. “Normal infections” include infection of chicks through an oral route and is characterized by a translocation through the intestinal epithelial cells followed with a splenic infection [2]. While asymptomatic carriers can be infected by *Salmonella* Enteritidis (SE) and *Salmonella* Typhmurium (ST), these bacteria can survive in the gastrointestinal tract of birds for months without showing clinical signs [3]. These *Salmonella* carriers have an infected gastrointestinal tract without showing clinical signs while excreting high concentrations of *Salmonella* into the environment [3,4,5,6,7,8,9,10]. These healthy carriers can be a risk to affect other birds by horizontal transmission or affect newly hatched chicks.

Despite the importance of *Salmonella* as a human pathogen, relatively little is known about the host immune response or virulence mechanisms of persistent asymptomatic infections in the avian intestine. The most fundamental question to answer is how do these organisms manage to escape clearance for so long in the presence of the host immune response? Upon infection with *Salmonella*, an up-regulation of the innate inflammatory response is generated and is characterized by pro-inflammatory cytokines and granulocyte (heterophils in chickens) influx within hours [11,12,13,14]. Yet, this intestinal inflammatory response is somehow dampened facilitating pathogen survival and persistent infection [15] for up to 10 weeks or more [3,16]. One must keep in mind that this dampened inflammatory response in chickens may be a host-developed mechanism to minimize immune-mediated damage to the intestine at a time when the gut microbiome is being established (disease tolerance). Disease tolerance has recently been described as a “distinct host defense strategy” [17,18,19,20,21,22]. Thus, a diminished immune response provides conceivable advantages to both the host and bacterium during a persistent infection in chickens. The mechanisms involved in this down-regulation of the mucosal immune response are currently unknown. However, one can speculate that mucosal disease tolerance is required to establish a persistent infection. During host tolerance, defined as coping with a pathogenic encounter without a consequent reduction in health [17,18,19,20,21,22], the host’s strategy is to avoid a harmful excessive inflammatory response [23,24]. However, this strategy may enable pathogen persistence, such as that observed with *Salmonella* infections of poultry [15,16].

We, and others, have recently demonstrated the development of a Th2, anti-inflammatory response in the cecum of chickens that begins at least four days after an initial infection with *Salmonella* and continues for weeks [11,25,26,27,28]. Moreover, we have noted a significant increase in CD4^+^CD25^+^ (T regulatory) cells in the cecum that corresponds to this shift from a pro-inflammatory to an anti-inflammatory environment [29]. T regulatory cells (Tregs) have been linked to play crucial roles sustaining a balance between the host immune response and immunological tolerance in many infections in mammals [30,31,32,33,34]. Further, a role for Tregs during a persistent *Salmonella* infection was recently described using a mouse model of persistence [35]. More recently, we also found alterations in the tissue phenotype of the cecum of the *Salmonella*-infected animals that is distinguished by metabolic signatures indicative of metabolic reprogramming with a shift from anabolic to catabolic reactions [28]. It is during this phase that we speculate that *Salmonella* takes advantage of a reduction of host response to infection to begin to establish a persistent cecal colonization [28].

In this study, our hypothesis was that *Salmonella enterica* serovar Enteritidis (*S*. Enteritidis) induces a disease tolerance host defense mechanism in chickens that allows the bacteria to colonize persistently the cecum of poultry. To test the hypothesis, we analyzed a time-course of chicken-specific kinomic immune changes and interferon-γ (IFN-γ) mRNA transcription in avian cecal tissue during a persistent infection by *S*. Enteritidis. Using these techniques, we were able to identify specific phosphorylation based immune post-translational signaling changes during a chronic *Salmonella* colonization in chickens that provide confirmation for the transition from an early mucosal pro-inflammatory response to the development of a disease tolerant mucosal response.

## 2. Results

### 2.1. S. Enteritidis Infection

Infection state of the chickens was confirmed by culturing the cecal contents and feces from each bird for *S*. Enteritidis with and without enrichment. Cultures showed that at least 75% of the chickens in the inoculated group were culture positive for *S*. Enteritidis throughout the experiment while *Salmonella* was never isolated from the birds in the control group at any given time point (Table 1 and Table 2). Four birds from each group at each time point were selected, infected birds were selected based on a consistent high level of *S.* Enteritidis colonization.

### 2.2. Kinome Arrays

Chicken-specific kinome arrays custom-designed for the study of chicken immune signal transduction pathways were used [36]. Kinome analysis was carried out on the cecal samples from non-infected and infected chickens. The results from four animals from each group (*S.* Enteritidis-infected and non-infected) and time point were combined to provide a representative result. To remove any non-specific or baseline phosphorylation signal from the analysis data from each time point was corrected using the matched uninfected controls. The kinome data were subjected to pathway overrepresentation analysis to determine which cellular pathways/processes are activated under the infected and non-infected conditions. To ensure that the identified pathways represent conserved and consistent biological responses, input data were limited to peptides with a consistent pattern of differential phosphorylation across the four biological replicates in at least one of the treatment sets as well as significant changes (*p* ≤ 0.05) in phosphorylation level relative to the non-infected control treatment. These select data from the four animals were merged to generate a representative data set for each treatment condition. All peptides that showed significant phosphorylation changes relative to control (*p* ≤ 0.05) for each time point were input into the Search Tool for the Retrieval of Interacting Genes/Proteins (STRING) database [37]. Using STRING functionality, Kyoto Encyclopedia of Genes and Genomes (KEGG) pathway results were generated for each dataset.

The STRING generated KEGG pathway results showed a number of pathways altered by the *S.* Enteritidis infection at a statistically significant level (*p* ≤ 0.05 false discovery rate (FDR) corrected). Of particular interest were those pathways that contained peptides that were significantly differentially phosphorylated at multiple times over the course of the study. A subset of these pathways are shown in Table 3. Of particular note, the T cell signaling and JAK-STAT signaling pathways were dramatically altered by the infection. Both these pathways had multiple peptide phosphorylation events altered at multiple time points post-infection. In total 49 differentially phosphorylated peptides were observed in chickens within these two different pathways on the 4th day post-infection with *S.* Enteritidis (Table 3) signifying a dramatic local post-translational modification of the proteins within the infected cecum. Of the 49 peptides that were differentially phosphorylated, 26 belong to the T cell signaling pathway, and 23 to the JAK-STAT pathway. Only 33 total peptides were found to be differentially phosphorylated over days 7–14 post-infection within these two specific pathways (Table 3).

### 2.3. Phosphoryaltion Events within Specific Pathways

#### 2.3.1. T Cell Receptor Signaling Pathway

The transcription factor family Nuclear factor of activated T-cells (NFAT), play a crucial role in regulating the transcription of cytokines and other genes critical for immune response. Members of the NFAT family were found to be significantly phosphorylated in the ceca of *S*. Enteritidis-infected chickens (Table 4). This is a significant finding because inactivated NFAT proteins in the cytoplasm of a cell are in their phosphorylated form. Following T cell receptor (TCR) stimulation, cytoplasmic NFAT proteins are dephosphorylated and translocate from the cytoplasm to the nucleus where they regulate transcription of key cytokine genes. Thus, based on the findings here the increased phosphorylation of NFAT inactivates the proteins preventing its translocation to the nucleus and thus decreasing pro-inflammatory cytokine production. Simultaneously, we found that Iκκ-β, NF-κB1 and NF-κB1A were significantly dephosphorylated after 4–10 days of infection with *S.* Enteritidis (Table 4). NF-κB is a transcription factor that is phosphorylated when activated by various intra- and extra-cellular stimuli then translocates into the nucleus and stimulates the expression of genes involved in a variety of immune functions.

Further analysis of the T cell receptor signaling pathway revealed two other significant changes in phosphorylation events: (1) phospholipase C-γ1 (PLCG1) was significantly dephosphorylated in the *S*. Enteritidis-infected cecal tissue at four days post-infection when compared to the non-infected control cecal tissue; and (2) a significant dephosphorylation of MAPKs, including MEK1, ERK1, MAP3K8, and p38.

#### 2.3.2. JAK-STAT Signaling Pathway

The JAK-STAT signaling cascade is represented quite comprehensively on the kinome array, it is possible to investigate the effects of a persistent cecal infection by *S*. Enteritidis on the principle signaling mechanism for a wide variety of cytokines and growth factors. At 4 days-post-infection, a differentiated series of phosphorylation events occurred at the receptor level in the infected birds when compared to the non-infected control birds (Table 5). First, a significant increase in phosphorylation of the IFN-α receptor (IFNAR1; *p* ≤ 0.003), and IL-2 receptor IL-2RB; (*p* ≤ 0.0003) were found. Simultaneously, there is a significant decrease on the phosphorylation of the IL-4 receptor (IL-4R; *p* ≤ 0.006), IFN-γ receptor (IFNGR1; *p* ≤ 0.006), IL-6 receptor gp130; (*p* ≤ 0.01), and IL-7 receptor (IL-7R; *p* ≤ 0.0008). Lastly, there was a five-fold increase in the phosphorylation of the IL-10 receptor (IL-10R-A; *p* ≤ 0.02) at four days post-infection was also elevated at 10 days post-infection (Table 5).

Furthermore, the development of a persistent cecal infection in chickens by *S.* Enteritidis also appears to target the JAK kinases for degradation. However, JAK2 and JAK3 appeared to be targets for dephosphorylation where both had a three-fold decrease in phosphorylation at four days post-infection (Table 5). JAK3 appears to be a specific target since the dephosphorylation continued through day 10 post-infection where an 18-fold decrease was observed.

Lastly, the persistent infection by *S*. Enteritidis also appeared to target the specific JAK substrate STAT4 (Table 5). We measured a significant two-fold (four days) to seven-fold (10 days) decrease in the phosphorylation of the STAT4 transcription factor in the ceca of the infected birds when compared to the cecal tissues from the non-infected birds. STAT1, 3, 5B and 6 had increased phosphorylation on day four post-infection, but all had a reduced phosphorylation 10–14 days post-infection.

### 2.4. Validation of Kinome Analysis with Antibody Array

An often used methodof validating kinome peptide array data is by using phosphospecific antibodies. For example, performing a Western blot using phosphospecific antibodies that correspond to the phosphosites on the peptide array. If the phosphospecific antibody binds and the peptide array shows the same phosphosite has been phosphorylated there is confirmation of the array data. This type of validation is similar to how transcriptome data from a cDNA microarray is validated through the use of quantitative real-time PCR. In a variation of the standard validation procedure we chose to employ an antibody microarray, which contains many phosphospecific antibodies immobilized in an array format [27]. Though there is a scarcity of chicken specific antibodies, many of the central proteins of interest found in the peptide array results were relatively well conserved between humans and chickens, providing confidence that there would be significant observed binding through cross-reactivity of the antibodies. To illustrate the conservation of phosphosites the percent orthology between the chicken and human 15 amino acid phosphorylation target sites determined by NCBI Protein Blast analysis is shown in Table 6. Following the data normalization, the results showed similar peptide phosphorylation events to those observed with the peptide arrays (Table 6).

### 2.5. Altered Expression of IFN-γ Transcription

**Figure 1:**
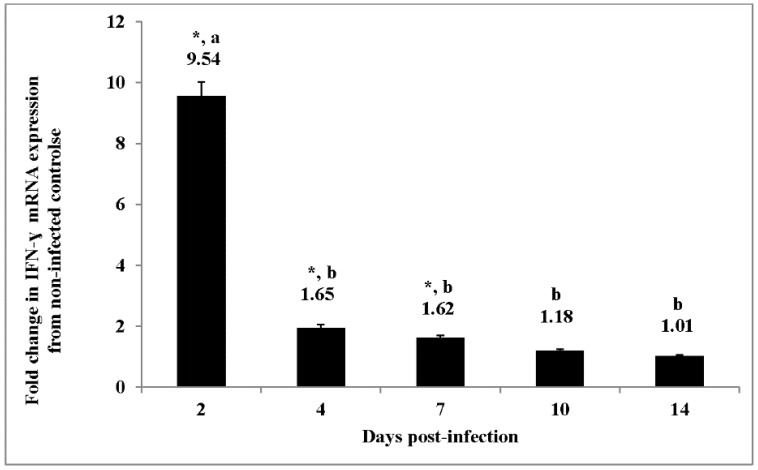
Expression of IFN-γ mRNA in the ceca from experimental chickens with persistent colonization by *Salmonella* Enteritidis. The expression of IFN-γ mRNA expression was determined by quantitative RT-PCR. Data represent the fold-change in mRNA expression in the cecum from infected chickens when compared to the mRNA expression in the cecum from non-infected chickens. Data represent the mean ± SEM from three separate experiments. * = significantly different from the non-infected controls. Different lower case letters = significantly different from infected chickens at 2 days post-infection (*p* ± 0.05).

As has been reported previously, during the early acute infection (within 24 h) by paratyphoid strains of *Salmonella* chickens up-regulate pro-inflammatory cytokines mRNA expression in the cecum [9,38,39,40]. In the present studies, we profiled the IFN-γ mRNA expression in the cecum of chickens 2, 4, 7, 10, and 14 days post-infection with *S*. Enteritidis and compared the results to the non-infected control birds. IFN-γ mRNA expression in the *S*. Enteritidis infected ceca from chickens was up-regulated two to seven days post-infection when compared to the non-infected birds expression in the cecum (Figure 1). However, there was a significant and dramatic nine-fold decrease in IFN-γ mRNA expression from day two post-infection to day four post-infection. The fold-change in IFN-γ mRNA expression remained unchanged through day 14 post-infection (Figure 1).

## 3. Discussion

Relatively little is known about how and why *Salmonella enterica* persist in the avian intestine, specifically the interactions between the virulence mechanisms and host immune response. The persistent colonization of the gut, the carrier state, is established, and the *Salmonella* is able to stay in the ceca for months without triggering clinical signs of infection [4,5,7,8]. A persistent, chronic, subclinical *Salmonella* infection of the intestinal tract is important to continued bacterial propagation and the contamination of poultry as it is nearly impossible to detect and isolate infected birds [41]. We, and others, have speculated that the bacterium is involved in redirecting, or subverting, the host response toward disease tolerance [11,26,27,28,42]. The present study was designed to address the question of immune tolerance induction during a persistent paratyphoid *Salmonella* infection in chickens.

Host responses to infectious agents are often regulated through phosphorylation. However, proteomic mechanisms of *Salmonella* acute infection biology and host responses to the bacteria have been investigated only in murine models [43,44,45,46,47]. Until recently, studies in poultry have been limited to the genomic responses of the host to infection (reviews in [11,48,49]). Our recent development of chicken-specific peptide arrays for kinome analysis of host phosphorylation-based cellular signaling responses provided us with the opportunity to develop a more detailed understanding of the chicken host-pathogen interactions with *Salmonella* [50,51].

Based on the findings here, our kinomic analysis demonstrate a phenotypic change in the avian cecum as it orchestrates the dynamics of immune signaling pathways, cytokine secretion, transcription factor expression, and the launch of a different immune microenvironment during the establishment of a persistent *Salmonella* infection and a return to intestinal homeostasis. Four days post-infection (pi) *Salmonella* induces an immune transition from an acute pro-inflammatory response to an established infection and a dampened or eliminated innate response [52]. By 4 days pi, we have described a substantial down-regulation of the expression pro-inflammatory cytokines that coincides with the up-regulation of the expression of anti-inflammatory cytokines [27,28]. Further, by day four pi a dramatic increase in Tregs (CD4^+^CD25^+^) in the cecum and remains elevated through 14 days pi [29]. This coordinated production of pro- versus anti-inflammatory responses is a central mechanism of an effective early inflammatory response and later return to tissue immune homeostasis. Finally, we used a kinomics approach to uncover the mechanisms used by *S*. Enteritidis to impact the avian inflammatory responses and determine host signaling events altered by the bacteria to create the conditions for a persistent infection. Our results identified multiple changes to the host kinome during the establishment of a persistent *Salmonella* infection in the avian cecum. This immune analysis that compared the immune responses between the *S*. Enteritidis-infected avian cecum and non-infected cecum provides novel information on host cellular signaling cascades that are altered during the establishment of *Salmonella* persistence (Table 5 and Table 6) [27,28]. Additionally, the relative lack of differential phosphorylation events found in the signaling pathways between the infected and non-infected ceca 7–14 days pi indicate that a level of immune homeostasis had been achieved and that the *Salmonella* were no longer being recognized as infectious agents and were now part of the commensal population. Further experiments are underway to further characterize and contrast this homeostasis to that of the non-infected controls.

Here we have further described a series of phosphorylation-mediated changes in the ceca of chickens during the development of a persistent *Salmonella* infection. The most significant differences in host immune kinase activities in infected animals occurred within four days pi. These changes were localized to select pathways, specifically the T cell receptor and the JAK-STAT signaling pathways, which were altered by the persistent colonization of the cecum by *S*. Enteritidis.

Stimulation of the T cell receptor results in the activation of the TCR signal transduction pathway. This pathway activates the transcription factors nuclear factor κB (NF-κB), nuclear factor of activated T-cells (NFAT), and activator protein 1 (AP-1), that induce expression of cytokine genes [53]. The results of this study clearly point to changes in the activity of all three of the central transcription factors, specifically at 4 days pi (Table 4). First, we found no significant effect on the phosphorylation of the AP-1 transcription factors between the *S*. Enteritidis-infected and non-infected tissues. The AP-1 pathway is dependent on activated of mitogen-activated protein kinases (MAPKs), such as extracellular signal–regulated kinase (ERK), c-Jun N-terminal kinase (JNK), and p38, which promote the synthesis, phosphorylation, and activation of the Fos and Jun proteins that together comprise the AP-1 transcription factor [53]. However, we found a significant dephosphorylation of MAPKs, including MEK1, ERK1, MAP3K8, and p38 involved in the T cell receptor signaling cascade; thus, pointing to the lack of involvement of AP-1-induced genes during a persistent *S*. Enteritidis infection in the chicken (Table 4). Second, NFAT (phosphorylated) and NF-κB (dephosphorylated) were significantly differentially phosphorylated in the ceca of *S*. Enteritidis-infected chickens (Table 4). The central question is whether there is a common thread that could account for this differential response of these transcription factors. This thread appears to be phospholipase C-γ1 (PLCG1) that was significantly dephosphorylated in the *S*. Enteritidis-infected cecal tissue at 4 days pi when compared to the non-infected control cecal tissue. Activation of both NF-κB and NFAT requires the activity of PLC-γ1, which generates the second messengers diacylglycerol (DAG) and inositol 1,4,5-trisphosphate (IP_3_). DAG leads to activation of protein kinase C θ (PKCθ), which, in turn, activates the inhibitor of κB (IκB) kinase (IKK) complex, resulting in the phosphorylation and degradation of IκBα and the translocation of the NF-κB p50:p65 heterodimer to the nucleus [54]. IP_3_ induces an increase in the concentration of cytoplasmic calcium (Ca^2+^) and activation of the Ca^2+^-dependent phosphatase calcineurin, which results in the rapid activation of NFAT, which is followed by its translocation to the nucleus [55]. To our knowledge, our report is the first to implicate *Salmonella* targeting of PLCG1 to manipulate the NF-κB and NFAT pathways to inhibit pro-inflammatory responses. What bacterial factors may be involved in dephosphorylating PLCG1 are unknown at this time and will be the focus of future experiments.

NFAT proteins, a family of transcription factors, are critical to the transcription of cytokine genes and other genes that are critical for the control of inflammation and regulation of the immune response [55,56]. Further, NFAT must ultimately bind to additional transcription factors, such as AP-1 to form transcriptional complexes that regulate gene expression that are inducibly transcribed by immune-system cells [55,57]. NFAT functions to regulate the interaction of the innate immune cells with acquired immunity and to promote anti-inflammatory programs (reviewed by [58]). Thus, the increased phosphorylation of NFAT peptides would suggest the initiation of anti-inflammatory signals. NF-κB is a transcription factor whose activity is triggered in response to infectious agents and pro-inflammatory cytokines via the IκB kinase (IKK) complex and plays a key role in regulating the pro-inflammatory response [59,60]. Therefore, dephosphorylation of both IKK and NF-κB would result in a down-regulation of pro-inflammatory cytokines as we observed in the present experiments. As a result, the T cell receptor signaling pathway analysis data provide evidence that the establishment of a persistent infection by *S*. Enteritidis in the avian cecum appears to be partially due to the targeting of signaling cascades that inhibit the transcription of pro-inflammatory responses and induce the beginning of a transition from T_H_1/T_H_17 cells to the development of Tregs [61,62,63,64].

Further pathway analysis of the kinome data indicated differential phosphorylation of the JAK-STAT pathway, a signaling cascade that provides a direct mechanism to translate an extracellular signal into a transcriptional response, in *S*. Enteritidis-infected cecal tissue. The JAK-STAT system consists of three main components: (1) a receptor; (2) Janus kinase (JAK); and (3) Signal Transducers and Activator of Transcription (STAT) [65]. Based on the results from them present experiments (Table 6), the IFN-α, IL-2, IL-4, and IL-10 receptors were phosphorylated; whereas, IFN-γ, IL-7, and IL-6 cytokine family (gp130) receptors were dephosphorylated. IFN-γ is characteristic of a Th1 response whereas IL-4 is a signature cytokine of Th2 responses. IL-7 is involved in early T cell development and IL-6 is a pro-inflammatory cytokine involved in stimulating an immune response during infection. IL-2 is normally produced by T cells during an immune response and involved in growth, proliferation, and differentiation of T cells to become “effector” T cells [66,67]. When combined with the cytokine expression observed previously [27,28], the down-regulation of IFN-γ mRNA transcription shown here (Figure 1) provides a clear pattern of down-regulation of the pro-inflammatory cytokines (IL-6, IL-1β, IFN-γ) and an up-regulation of anti-inflammatory cytokines, IL-10 and TGF-β4 [27,28]. We speculate there is a profound immune transition from an active inflammatory response where the immune system was working to reduce the number of bacteria to an environment of homeostasis where the immune response is allowing for a persistent state of infection in the *S*. Enteritidis-infected cecal tissue.

Cytokine receptor proteins lack enzymatic activity, thus are dependent upon JAKs to initiate signaling upon binding of their ligands. The JAK family has four members: JAK1, JAK2, JAK3 and tyrosine kinase 2 (TYK2) [68]. TYK2 is the only JAK family member that was activated (phosphorylated) in the ceca from the *S*. Enteritidis-infected chickens (Table 6). Although primarily involved in IL-12 and type I-IFN signaling, TYK2 is activated by IL-10 [69].

Most importantly, based on these experiments, the development of a persistent cecal infection by *S*. Enteritidis triggers a dephosphorylation of both JAK2 and JAK3 proteins (Table 6). JAK2 is an essential tyrosine kinase for modulating the immune response and whose activation contributes to the severe inflammatory response in sepsis [70,71]. Inhibition of JAK2 prevents NF-κB activation; thus “rescuing” mice from polymicrobial sepsis [72]. Therefore, we can conclude that the dephosphorylation of both JAK2 and NF-κB found via our kinomic analysis is indicative of a negative regulation of a pro-inflammatory response; in this case brought about by the establishment of a persistent *Salmonella* infection. Further experiments are required to confirm this hypothesis.

JAK3 is predominantly expressed in hematopoietic lineage such as NK cells, T cells and B cells and intestinal epithelial cells [73,74,75]. JAK3 is the only JAK family member involved in all phases of T cell biology: development, proliferation, and differentiation [76,77,78]. For T cell differentiation, JAK3, along with IL-4, steer Th2 cell differentiation [78], but inhibition of JAK3 generates the induction of Tregs [79,80]. Therefore, the dephosphorylation of JAK2 and JAK3 found in the present studies would result in a change in the functional immune phenotype of the cecal environment that benefits the establishment of a tolerant mucosal immune response against the bacterial colonization. Although these studies cannot confirm what provoked this dephosphorylation of JAK2 and JAK3, we speculate that the mechanism is a specific action of the *Salmonella* organism as it begins to establish its long term colonization. These results are the first to infer that *Salmonella* have evolved a time-dependent strategy that blocks responsiveness of the JAK proteins that down-regulates the host response to infection.

STAT4 is a decisive factor in host resistance to a variety of viral, bacterial, and protozoan pathogens while serving as the central regulator of IFN-γ production during inflammation [81]. Intestinal IFN-γ mRNA expression levels are a prevailing indicator of a reduced immune response associated with persistence of *Salmonella* in the chicken gastrointestinal tract [42]. Furthermore, the ratio between STAT1 and STAT4 are crucial for IFN-γ production during viral and *Salmonella* infections [82,83]. Herein, we found a reduced IFN-γ mRNA expression during the establishment of the persistent *Salmonella* infection (Figure 1) and an increased phosphorylation of STAT1 and dephosphorylation of STAT4 (Table 6). Our results are in agreement with two recent studies where *N*-ethyl-*N*-nitrosourea-induced mutations of mice resulted in increased STAT1 phosphorylation, suppressed STAT4 expression, and altered IFN-γ production that led to the increased susceptibility of the animals to *S*. Typhimurium infection [83,84]. IFN-γ has been shown to play a fundamental role in the resolution of intestinal Salmonella infection [13,42,85]. Further, our observation of a dramatic decrease in IFN-γ mRNA expression at day four p.i. is in agreement with previously reported results by other laboratories [13,26].

## 4. Materials and Methods

### 4.1. Experimental Animals

Experiments were conducted according to the regulations established by the United States Department of Agriculture Animal Care and Use Committee. Broiler chickens used in this study were obtained from a commercial breeder and were all of the same genetic background and were not vaccinated at any time. Chicks were placed in floor pens containing wood shavings, provided supplemental heat, water, and a balanced, unmedicated corn and soybean meal-based chick starter diet ad libitum that met or exceeded the levels of critical nutrients recommended by the National Research Council [86]. *Salmonella* was not detected in the feed or from the paper tray liners.

### 4.2. S. Enteritidis Challenge

A poultry isolate of *Salmonella enterica* serovar Enteritidis (*S*. Enteritidis; (ID 9711771, part 24)) was obtained from the National Veterinary Services Laboratory (Ames, IA, USA), and was selected for resistance to nalidixic acid and novobiocin and maintained in tryptic soy broth (Difco Laboratories, Sparks, MD, USA) containing antibiotics (20 µg/mL nalidixic acid and 25 µg/mL novobiocin; Sigma Chemical Co.; St. Louis, MO, USA). A stock culture was prepared in sterile PBS and adjusted to a concentration of 1 × 10^9^ colony forming units (CFU/mL). The viable cell concentration of the challenge dose for each experiment was determined by colony counts on XLT4 agar base plates with XLT4 supplement (Difco) and nalidixic acid and novobiocin (XLT-NN).

### 4.3. Experimental Design

One-day-old broiler chickens were randomly distributed into two experimental groups: non-infected control and infected. Each group contained 200 birds fed a balanced, unmedicated corn and soybean meal-based diet. Four days post-hatch, all chickens were orally challenged with 1 mL of either 5 × 10^6^ CFU/mL *S*. Enteritidis or mock challenged with 1 mL sterile PBS. Four, 7, 10, and 14 days after challenge, 50 chickens from each group were killed by cervical dislocation, cecal contents were analyzed for *S*. Enteritidis colonization, 10 of these chickens were used for: (a) cecal tonsils for quantitative real-time PCR (qRT-PCR); and (b) cecal tissue was flash frozen in liquid nitrogen and stored for use in the peptide and antibody arrays.

All experiments were conducted three times. Therefore, the ceca from a total of 30 chickens for each of the 2 groups (10 chickens each in 3 experiments) were used to prepare the mRNA for the qRT-PCR IFN-γ assay described below. RNA from each bird (*n* = 10) was isolated and assayed separately and not pooled. Each RNA sample was replicated 3 times for IFN-γ expression per experiment.

### 4.4. Sample Collection for Peptide and Antibody Arrays

At 4, 7, 10, and 14 days post infection, both ceca were removed from each of 10 birds from each group (non-infected and infected) and immediately flash frozen in liquid nitrogen to preserve kinase enzymatic activity. Samples were taken from liquid nitrogen and transferred to a −80 °C freezer until further experimental procedures were conducted.

### 4.5. Kinome Array

At each of the time points and under each condition (infected and uninfected), 4 cecal samples from 4 different animals were taken from storage for analysis (32 samples total). Infected birds were selected based on a consistent high level of *S.* Enteritidis colonization. Cecal tissue samples were weighed to obtain a consistent 40 mg sample for the array protocol. Samples were homogenized by a hand-held Qiagen TissueRuptor (Valencia, CA, USA) in 100 μL of lysis buffer (20 mM Tris–HCl pH 7.5, 150 mM NaCl, 1 mM EDTA, 1 mM Ethylene glycol tetraacetic acid (EGTA), 1% Triton X-100, 2.5 mM sodium pyrophosphate, 1 mM Na_3_VO_4_, 1 mM NaF, 1 μg/mL leupeptin, 1 g/mL aprotinin and 1 mM Phenylmethylsulphonyl fluoride (all products from Sigma Aldrich (St. Louis, MO, USA), unless indicated). Following homogenization, the peptide array protocol was carried out as per Jalal et al. [87], with alterations described in Arsenault et al. [51,88].

### 4.6. Antibody Array

The antibody array assay kit was procured from Full Moon BioSystems (Sunnyvale, CA, USA). This technique was used as an alternative to procuring phosphospecific antibodies individually and performing several western blot assays. The protocol was carried out as per manufacturer’s instructions (Antibody Array User’s Guide Rev 11.3) with the following alteration to the homogenization step: instead of using the bead and vortex homogenization indicated in the kit, the hand-held Qiagen Tissue Ruptor was used.

### 4.7. Data Analysis: Kinome and Antibody Arrays

Data normalization and PCA analysis was performed for both the peptide and antibody microarrays as per Li et al. [89] using the PIIKA2 online platform (http://saphire.usask.ca/saphire/piika/index.html). Briefly, the array data were analyzed by subtracting the background intensity from the foreground intensity, variance stabilization normalization was conducted to bring all of the arrays onto the same scale, and then *t*-test, clustering and pathway analysis were performed. This consistent analysis method facilitated a more direct comparison between the two distinct array datasets and allowed for a statistically robust analysis of the phosphorylation events being measured. Geneontology (GO) and Kyoto Encyclopedia of Genes and Genomes (KEGG) pathway analysis was performed by uploading the statistically significant peptide lists to the Search Tool for the Retrieval of Interacting Genes (STRING) [36].

### 4.8. Sample Collection for Bacterial Contents

The ceca from each chicken was removed aseptically, and the contents (0.25 g) were serially diluted to 1:100, 1:1000, or 1:10,000 and spread onto XLT-NN plates. The plates were incubated at 37 °C for 24 h, and the number of NN-resistant *S*. Enteritidis cells per gram of cecal contents was determined. The data from each experimental group were pooled from three separate trials for statistical analysis.

### 4.9. Sample Collection for mRNA

Chickens from each experimental group were euthanized at 4, 7, 10, and 14 days post-infection. A 25-mg piece of tissue was removed from the cecal tonsils. The tissue was washed in PBS and placed in a 2-mL microcentrifuge tube with 1 mL of RNAlater (Qiagen, Inc., Valencia, CA, USA) and stored at −20 °C until processed.

### 4.10. RNA Isolation

Tissues (50 mg) were removed from RNAlater and transferred to pre-filled 2 mL tubes containing Triple-Pure™ 1.5 mm zirconium beads. RLT lysis buffer (600 μL) from the RNeasy mini kit (Qiagen, Valencia, CA, USA) was added and the tissue was homogenized for 1–2 min at 4000 rpm in a Bead Bug microtube homogenizer (Benchmark Scientific, Inc., Edison, NJ, USA). Total RNA was extracted from the homogenized lysates according to the manufacturer’s instructions, eluted with 50 μL RNase-free water, and stored at −80 °C until qRT-PCR analyses performed. RNA was quantified and the quality was evaluated using a spectrophotometer (NanoDrop Products, Wilmington, DE, USA). The data from these three repeated experiments were pooled for presentation and statistical analysis. Total RNA (300 ng) from each sample was prepared.

### 4.11. Quantitative Real-Time PCR

The primer and probe sets for IFN-γ and 28S rRNA were designed using the Primer Express software program (Applied Biosystems, Foster City, CA, USA). IFN-γ mRNA expression was quantitated using a well-described method. Primers and probes for IFN-γ and 28S rRNA-specific amplification have been described [25,47] and are provided in Table 7. The qRT-PCR was performed using the TaqMan fast universal PCR master mix and one-step RT-PCR master mix reagents [27,28] (Applied Biosystems). Amplification and detection of specific products were performed using the Applied Biosystems 7500 Fast real-time PCR system with the following cycle profile: one cycle of 48 °C for 30 min and 95 °C for 20 s and 40 cycles of 95 °C for 3 s and 60 °C for 30 s. Quantification was based on the increased fluorescence detected by the 7500 Fast sequence detection system due to hydrolysis of the target-specific probes by the 5 = nuclease activity of the r*Tth* DNA polymerase during PCR amplification. Normalization was carried out against 28S rRNA, which was used as a housekeeping gene. To correct for differences in RNA levels between samples within the experiment, the correction factor for each sample was calculated by dividing the mean threshold cycle (*C*_t_) value for 28S rRNA-specific product for each sample by the overall mean *C*_t_ value for the 28S rRNA-specific product from all samples. The corrected cytokine mean was calculated as follow: (average of each replicate × cytokine slope)/(28S slope × 28S correction factor). Fold changes in mRNA levels were calculated from mean 40 *C*_t_ values by the formula 2^(40 *C*t infected group − 40 *C*t in non-infected control)^.

### 4.12. Statistical Analysis: mRNA Expression

The mean and standard error of the mean were calculated and differences between groups were determined by analysis of variance. Significant differences were further separated using Duncan’s multiple range test [27]. Fold changes in RNA levels were calculated from mean 40 *C*_t_ values using formula 2^(40 *C*t infected group − 40 *C*t in non−infected control)^. A *p* value of ≤0.05 was considered statistically significant.

## 5. Conclusions

Collectively, we have outlined a series of altered phosphorylation events in multiple signaling pathways in the cecum of *S*. Enteritidis-infected chickens that induces an immunological tolerogenic response beginning around three to four days post-primary infection. The tolerance is characterized by alterations in T cell signaling pathway and blockage of IFN-γ protection through the disruption of the JAK-STAT signaling pathway. Further, the tolerance response induces a reduction in pro-inflammatory cytokine mRNA expression and an increase in anti-inflammatory cytokine mRNA expression.

## Figures and Tables

**Table 1:** Number of chickens positive for *Salmonella* Enteritidis ceca colonization for 2 weeks following challenge.

Treatment Groups	Percent Positive for *Salmonella* Enteritidis Cecal Colonization (Total Positive/Total Challenged)			
	Days post-challenge			
	4	7	10	14
Non-infected control	0	0	0	0
	(0/50)	(0/50)	(0/50)	(0/50)
Infected	100	100	90	83
	(50/50)	(50/50)	(45/50)	(41/50)

**Table 2:** Cecal *Salmonella* Enteritidis CFUs for 2 weeks following challenge.

Treatment Groups	CFU of *Salmonella* Enteritidis in Cecum (log _10_)			
	Days post-challenge			
	4	7	10	14
Non-infected control	0	0	0	0
Infected	5.398 ± 1.112	5.708 ± 1.341	4.342 ± 00.859	3.476 ± 1.472

**Table 3:** KEGG Pathways generated by Search Tool for the Retrieval of Interacting Genes/Proteins (STRING).

		4 Days		7 Days		10 Days		14 Days		
GO ID	Pathway	# Peptides	*p*-Value (FDR)	# Peptides	*p*-Value (FDR)	# Peptides	*p*-Value (FDR)	# Peptides	*p*-Value (FDR)	Reference
hsa04660	T cell receptor signaling pathway	26	3.09 × 10^−18^	-	N/S	9	2.9 × 10^−5^	11	6.93 × 10^−7^	Here
hsa05130	Pathogenic Escherichia coli infection	-	N/S	2	N/S	4	N/S	4	N/S	-
hsa04250	TGF-β4 signaling pathway	7	0.016	-	N/S	-	N/S	-	N/S	[27]
hsa04310	Wnt signaling pathway	13	0.0004	3	N/S	-	N/S	6	0.024	[27]
hsa05217	Basal cell carcinoma	-	N/S	-	N/S	2	0.338	-	N/S	-
hsa04150	mTOR signaling pathway	13	4.83 × 10^−10^	2	N/S	5	1.32 × 10^−3^	-	N/S	[28]
hsa04630	JAK-STAT signaling pathway	23	4.13 × 10^−12^	1	3.8 × 10^−2^	6	2.14 × 10^−4^	6	2.9 × 10^−4^	Here

Peptides that displayed a significant change in phosphorylation state were input into the STRING database for each time point. Generated pathways involved in immune activation/suppression that displayed *p*-value of less than 0.05 (FDR corrected) are listed. # Peptides refers to number of peptides within the given pathway that were present within the peptide array data set. N/S indicates that the pathway is non-significant.

**Table 4:** Peptides from the T cell receptor signaling pathway that displayed a statistically significant change in phosphorylation.

T Cell Receptor Signaling Pathway								
Days Post Infection								
	4		7		10		14	
Peptide	Fold Change	*p*-Value	Fold Change	*p*-Value	Fold Change	*p*-Value	Fold Change	*p*-Value
Akt1	1.52	0.03	-	-	-	-	-	-
Akt3	1.80	0.04	-	-	-	-	-	-
Cbl Y728	−1.97	0.03	-	-	-	-	−1.23	0.03
Cbl Y773	1.30	0.02	-	-	-	-	-	-
CDC42	1.69	0.01	-	-	-	-	-	-
IKK-β	−2.87	5.39 × 10^−5^	-	-	−1.91	0.04	-	-
FYN	-	-	-	-	−2.29	0.03	-	-
GRB2	-	-	-	-	−1.84	0.04	-	-
GSK-3β	−2.25	0.002	-	-	−2.45	0.02	-	-
HRAS	−1.97	0.009	-	-	-	-	-	-
ITK	−2.87	0.007	-	-	-	-	1.61	0.04
Jun S59	3.60	0.0008	-	-	-	-	-	-
Jun S69	−3.39	0.0004	-	-	-	-	-	-
MEK1	−3.23	0.03	-	-	-	-	-	-
MEK2	1.25	0.01	-	-	-	-	-	-
MAP2K2	−1.55	0.01	-	-	-	-	-	-
MAP3K14	2.87	0.005	-	-	-	-	1.10	0.03
MAPK3K7	2.71	0.02	-	-	4.48	0.01	-	-
MAP3K8	−1.40	0.02	−2.13	0.01	-	-	-	-
p38 MAPK (MAPK11)	−1.97	0.02	-		-	-	-	-
p38 MAPK (MAPK14)	-	-	-	-	-	-	−1.72	0.04
ERK1	−4.27	0.001	-	-	1.61	0.01	-	-
NFATC1	-	-	-	-		-	1.96	0.01
NAFATC2	1.78	0.04	-	-	-	-	-	-
NFATC3	2.24	0.01	-	-	-	-	-	-
NFκB1	−3.12	0.02	-	-	-		−1.65	0.02
NFκB1A	−2.59	0.001	-	-	−1.54	0.01	−1.57	0.01
PAK1 S198	−1.41	0.04	-	-	-	-	-	
PAK1 T212	5.36	0.001	-	-	-	-	1.05	0.02
PAK1 T422	−2.64	0.01	-	-	-	-	-	-
PI3KR1	3.08	0.02	-	-	-	-	-	-
PLCG1	−2.79	0.001	-	-	-	-	-	-
PRKCQ	−1.47	0.003	-	-	-	-	-	-
PTPRC	-	-	-	-	−2.97	0.02	-	-
RAF1	−2.29	0.003	-	-	-	-	1.36	0.01
SOS1	3.15	0.01	-	-	1.76	0.04	1.94	0.03

Peptides that displayed a *p*-value of less than 0.05 are listed.

**Table 5:** Peptides from the JAK-STAT signaling pathway that displayed a statistically significant change in phosphorylation.

	JAK-STAT Signaling Pathway							
	DAYS POST-INFECTION							
	4		7		10		14	
Peptide	Fold Change	*p*-Value	Fold Change	*p*-Value	Fold Change	*p*-Value	Fold Change	*p*-Value
AKT1	1.52	0.03	-	-	-	-	-	-
AKT3	1.80	0.04	-	-	-	-	-	-
Cbl	1.29	0.02	-	-	-	-	-	-
IFNAR1	1.88	0.003	-	-	-	-	-	-
IFNGR1	−1.53	0.01	-	-	-	-	-	-
IL-10R-A	5.10	0.03	-	-	2.72	0.03	-	-
IL-2RB	7.89	0.0003	-	-	-	-	-	-
IL4R	1.37	0.01	-	-	5.19	0.003	-	-
IL-6R	−1.81	0.01	-	-	-	-	-	-
IL7R	−6.10	0.001	-	-	-	-	-	-
Jak2	−3.11	0.004	-	-	-	-	−1.69	0.05
Jak3	−3.69	0.002	−1.68	0.02	−18.74	0.0006	-	-
PIK3R1	3.08	0.02	-	-	-	-	-	-
PIM1	−3.09	0.03	-	-	-	-	1.26	0.02
SOS1	3.15	0.01	-	-	1.76	0.04	1.65	0.003
STAT1	2.48	0.03	-	-	-	-	−2.17	0.009
STAT3 S728	2.44	0.03	-	-	-	-	1.94	0.03
STAT3 Y706	1.99	0.04	-	-	-	-	-	-
STAT4	−2.73	0.05	-	-	−7.52	0.04	-	-
STAT5B Y699	3.33	0.028599	-	-	-	-	-	-
STAT5B Y740			2.01	0.04	-	-	−1.29	0.01
STAT6	4.07	0.003	-	-	−1.58	0.007	-	-
TYK2	1.49	0.02	-	-	-	-	-	-

Peptides that displayed a *p*-value of less than 0.05 are listed.

**Table 6:** Antibody array results.

Antibody Array			Peptide Array			% Homology
ID	Fold Change	*p*-Value	ID	Fold Change	*p*-Value	
AMPK (Phospho-Thr174)	2.05	0.02	AMPK1 S173	4.23	0.03	100
ATF2 (Phospho-Ser112/94)	−2.13	0.02	ATF2 T72	−2.88	0.005	
Calmodulin (Phospho-Thr79/Ser81)	−1.48	0.01	Calmodulin T80	−1.53	0.04	100
			Calmodulin Y100	−1.56	0.003	
CAMK2-β/γ/Δ (Phospho-Thr287)	1.17	0.01	CAMK2-alpha T305	2.79	0.01	100
CDC25C (Phospho-Thr48)	−1.54	0.03	Cdc25A T510	−1.99	0.002	100
Ezrin (Phospho-Thr566)	2.14	0.03	Ezrin Y477	2.53	0.02	100
FAK (Phospho-Ser910)	2.04	0.03	FAK Y397	4.18	0.04	100
FLT3 (Phospho-Tyr842)	−1.20	0.02	FLT3 Y452	−1.81	0.03	79
HSP27 (Phospho-Ser15)	1.12	0.02	HSP27 S15	−4.35	0.01	67
c-Jun (Phospho-Tyr170)	4.27	0.04	Jun S59	3.60	0.001	85
MEK1 (Phospho-Thr291)	−1.22	0.03	MEK1 S222	−3.23	0.04	100
MEK-2 (Phospho-Thr394)	−1.58	0.04	MEK2 S220	−1.55	0.01	100
MSK1 (Phospho-Ser376)	−1.28	0.01	MSK1 S366	−3.26	0.008	100
P38 MAPK (Phospho-Thr180)	−1.39	0.05	P38-alpha Y181	−1.97	0.02	100
PAK1 (Phospho-Thr122)	1.44	0.04	PAK1 T212	5.36	0.001	80
PKC delta (Phospho-Tyr52)	−1.36	0.04	PKCD Y311	−1.15	0.001	100
PLCG1 (Phospho-Tyr783)	−1.20	0.03	PLCG1 Y675	−2.79	0.001	83
SMAD 2 (Phospho-Thr220)	1.48	0.04	SMAD2 S245	3.43	0.01	100
			Smad2 S255	4.53	0.01	
Smad 2/3 (Phospho-Thr8)	1.09	0.03	Smad3 T180	1.44	0.03	
Src (Phospho-Tyr418)	−1.61	0.005	Src Y416	−1.44	0.03	100
			Src Y527	−2.12	0.02	
STAT3 (Phospho-Ser717)	1.98	0.03	STAT3 S728	2.44	0.03	100
			STAT3 Y706	1.99	0.04	
Trk (Phospho-Tyr515)	1.13	0.04	TrKA Y490	−1.52	0.02	84
			TrKA Y674	−1.68	0.03	
			TrKA Y785	−2.04	0.0	
XIAP (Phospho-Ser87)	1.17	0.04	XIZP S87	2.12	0.002	60

Statistically significant (*p* ≤ 0.05) phosphospecific antibody array results of *Salmonella* Entertidis cecal samples. Four days post-infection samples were compared to non-infected control samples to find changes in infected cecal tissue over time. Antibodies due to being bound to phosphorylated protein had a statistically significant difference in fluorescent signal are shown. Fold Change Antibody Array is the change in fluorescent signal when comparing the infected samples to control samples. Homology indicates the % similarity between human and chicken at the 15 amino acid region flanking the phosphorylation residue. Fold Change Peptide Array is the change in fluorescent signal as indicated by the peptide array. N/A indicates the exact phosphorylation target residue on the antibody array was not present on the peptide array or not significantly differentially phosphorylated.

**Table 7:** Real-time quantitative RT-PCR probes and primers for 28S and IFN-γ.

RNA Target		Probe/Primer Sequence	Accession Number ^a^
28S	Probe	^d^ 5′-(FAM)-AGGACCGCTACGGACCTCCACCA-(TAMRA)-3′	X59733
	F ^b^	5′-GGCGAAGCCAGAGGAAACT-3′	
	R ^c^	5′-GACGACCGATTGCACGTC-3′	
IFN-γ	Probe	^d^ 5′-(FAM)-TGGCCAAGCTCCCGATGAACGA-(TAMRA)-3′	YO7922
	F	5′-GTGAAGAAGGTGAAAGATATATCATGGA-3′	
	R	5′-GCTTTGCGTGGATTCTCA-3′	

^a^ Genomic DNA sequence; ^b^ Forward; ^c^ Reverse; ^d^ 5-carboxyfluorescein.
